# Metabolic profiling of Parkinson's disease: evidence of biomarker from gene expression analysis and rapid neural network detection

**DOI:** 10.1186/1423-0127-16-63

**Published:** 2009-07-13

**Authors:** Shiek SSJ Ahmed, Winkins Santosh, Suresh Kumar, Hema T Thanka Christlet

**Affiliations:** 1 Department of Biotechnology, School of Bioengineering, SRM University, Kattankulathur, Tamil Nadu, 603 203, India; 2 Department of Neurology, SRM Medical College Hospital & Research Centre, Kattankulathur, Tamil Nadu, 603 203, India

## Abstract

**Background:**

Parkinson's disease (PD) is a neurodegenerative disorder. The diagnosis of Parkinsonism is challenging because currently none of the clinical tests have been proven to help in diagnosis. PD may produce characteristic perturbations in the metabolome and such variations can be used as the marker for detection of disease. To test this hypothesis, we used proton NMR and multivariate analysis followed by neural network pattern detection.

**Methods & Results:**

^1^H nuclear magnetic resonance spectroscopy analysis was carried out on plasma samples of 37 healthy controls and 43 drug-naive patients with PD. Focus on 22 targeted metabolites, 17 were decreased and 5 were elevated in PD patients (p < 0.05). Partial least squares discriminant analysis (PLS-DA) showed that pyruvate is the key metabolite, which contributes to the separation of PD from control samples. Furthermore, gene expression analysis shows significant (p < 0.05) change in expression of *PDHB *and *NPFF *genes leading to increased pyruvate concentration in blood plasma. Moreover, the implementation of ^1^H- NMR spectral pattern in neural network algorithm shows 97.14% accuracy in the detection of disease progression.

**Conclusion:**

The results increase the prospect of a robust molecular definition in detection of PD through the early symptomatic phase of the disease. This is an ultimate opening for therapeutic intervention. If validated in a genuinely prospective fashion in larger samples, the biomarker trajectories described here will go a long way to facilitate the development of useful therapies. Moreover, implementation of neural network will be a breakthrough in clinical screening and rapid detection of PD.

## Background

Parkinson's disease (PD) is a slow, progressive degenerative disorder of the central nervous system [1]. It is characterized by muscle rigidity, tremor, slowing of physical movement (Bradykinesia) and, in extreme cases, loss of physical movement (Akinesia) [2]. The classification of the stages were done based on Unified Parkinson's Disease Rating Scale (UPDRS) [3] or on an equivalent rating scale value such as Hoehn and Yahr scale and Schwab and England Activities of Daily Living Scale. The UPDRS has been used extensively by researchers and clinicians around the world. The rating scale is categorized as stages based on bradykinesia, dyskinesias, rigidity, posture, swinging arms, walking, tremor, gesture, seborrhea, speech, sensory complaints, depression, postural stability, insomnia, leg agility and independence. The diagnosis is based on medical history and neurological examinations [4]. The current diagnosis of PD remains unclear, because of the complex spectrum of symptoms and their similarities with other neurodegenerative diseases, such as multiple system atrophy and progressive supranuclear palsy. Moreover, clinical diagnosis fails to identify PD before they cause a significant loss of dopamine neurons [5]. There is immense need for early detection [6] and more effective drugs for the cure of PD. An understanding of the molecular characteristics underlying the disease processes of PD is a prerequisite for the development of biomarker in the early detection for providing high value therapeutics.

Biomarkers serve as tools for diagnosis of any disease usually performed on readily accessible body fluids, such as cerebrospinal fluid (CSF), serum, urine or saliva. Metabolic profiling is one of the most important techniques, greatly focused for the detection of biomarkers for diagnosis of diseases [7,8]. Several encouraging results were obtained using metabolite profiling in an attempt to diagnose coronary heart disease [9], diabetes mellitus [10], eclampsia [11], lipid disorder [12], colon carcinoma [13], epithelial ovarian cancer [14], hypertension [15], kidney deficiency syndrome [16], motor neuron disease [17] and liver cancer [18]. Several techniques are presently available for metabolite profiling, such as nuclear magnetic resonance spectroscopy (NMR), mass spectrometry (MS), high-performance liquid chromatography (HPLC), gas chromatography (GC) and optical spectroscopic (OS) analysis. NMR spectroscopy technique is more ease for rapid metabolite detection because it requires an uncomplicated, preprocessing procedure, and it is a feasible method to obtain essential information from complex and intact biological samples. Recent studies show that NMR metabolic profiling plays a vital role in clinical diagnosis [19,20].

In the present study, ^1^H (proton) NMR spectroscopy was executed to examine the plasma samples of 37 normal and 43 drug-naive patients of PD to identify the metabolite variations of 22 targeted metabolites between the blood plasma of healthy individuals and patients.

## Materials and methods

### Clinical sample

Clinical samples were collected from the out-patient setting of the Department of Neurology at SRM Hospital, Tamil Nadu, India. Drug-naive samples of 43 PD patients were collected and 37 samples of age and gender-matched healthy controls were included for comparative study (Table 1), written consent was obtained from each participant during in-person interview and blood donation. Data on gender, age and weight were collected. For participants with PD, date of the symptom onset, date of diagnosis, and family history of PD were recorded. The ethical committee of SRM Medical College Hospital & Research, India reviewed and approved the protocol of this study (Ref. No.3496/Dean/07). The pathological status was well studied by neurological specialists of hospital and the disease stages were classified by UPDRS.

**Table 1:** Statistics

Sample Information	Number of samples	Statistics		Gender	
		
		Mean Age	SD	Male	Female
Normal	37	58.486	11.843	21	16

PD-Stage 1	15	58.2	11.651	9	6

PD-Stage 2	13	60.153	11.238	8	5

PD-Stage 3	15	55.66	12.056	8	7

Statistics of research participant's involved in the study.

### Preparation of samples

Blood samples (4 ml) were collected in EDTA vacutainer tube (Becton Dickinson, Franklin Lakes, NJ) from individuals and immediately centrifuged for 5 min at 14,000 rpm using eppendorf centrifuge to separate plasma from other cellular material. Subsequently, the plasma was transferred to fresh eppendorf tube and kept at -20°C before processing. 500 μl of plasma was processed by Nanosep 3KD (Pall Co., New York, USA) micro centrifuge tube to extract the metabolites from the sample and to avoid protein interference in the extract. The obtained metabolite extracts were dissolved in D_2_O (Merck KGaA, Darmstadt, Germany) and 500 μM of 2,2-dimethyl-2-silapentane-5-sulfonate (DSS) (Sigma Chemical Co., St. Louis, MO, U.S.A) were added as an internal standard in preparation for ^1^H-NMR analysis.

### Proton NMR spectrum

All experimental samples were subjected to ^1^H-NMR spectrum. The spectrum was acquired using the Bruker AV-III-500 MHz (Bruker Biospin, Inc., Milton, Canada) operating at 500.1323506 MHz and equipped with a five mm PABBO BB probe at 295K. A total of 231 scans was collected over a sweep width of 6066 Hz, with a 5s repetition time. Fourier transformation, phasing, line broadening and baseline correction was made prior to the analysis of metabolites. Chemical shift assignments were conformed by performing correlation spectroscopy (COSY) and total correlation spectroscopy (TOCSY) 2D NMR using standard Bruker pulse programs.

### Metabolite determination

To evaluate the metabolites variability between biofluid derived from patients and healthy controls, proton NMR spectra was analyzed using the novel Chenomx NMR 5.1 software (Chenomx. Inc., Edmonton, Canada). The Profiler module was used for the determination and quantification of 22 metabolites in the plasma by comparing with a library of 292 metabolites of ^1^H-NMR spectra of each standard compound recorded at 500 MHz. The calibration of chemical shift was made by DSS internal standard (0.0 ppm) resonance.

### Statistical analysis

The profiled data were imported into GeneSpring GX7.3 microarray software (Agilent Technologies Inc., Santa Clara, California), in which analysis of variance (ANOVA) was performed. In order to confirm the biomarkers differentiating the patients with PD from matched controls, partial least square discriminant analysis (PLS-DA) was employed using Umatrices software (Umetrics, Inc., Kinnelon, NJ).

### Systems biological approach

Pubgene index [21] was used to identify the interacting genes with pyruvate dehydrogenase complex. Moreover, pyruvate dehydrogenase is multi enzyme complex, which includes *PDHB, PDHA1, PDHA2, DLAT, DLD *and *PDHX *[22].

### Gene expression analysis

Secondary microarray data analysis was performed in the cellular blood of PD patients on previously published data retrieved from gene expression omnibus [GEO: GDS2519], NCBI database. The analysis was performed on 72 samples, which include 22 normal and 50 early PD samples [23]. Prior to analysis, the basic processing of raw data was carried out on 40 gene spots identified from the gene interactions, such as *PDHB, PDHA1, PDHA2, DLAT, DLD, PDHX, CAT, CD4, CD8A, CS, DUOX1, DUOX2, EDC4, FAM48A, FGF13, FGF, GCG, G6PD, GSR, HSPD1, IFNG, INS, INSR, JUN, NEUROG3, NOS1, NOS2A, NOS3, NPFF, OGDH, PDX1, PTPRC, SST, SYT1, THEG, TMEM16A, TNF, TTF2, UNC5C *and *WNK1*. The analysis was made only on these selected genes using GeneSpring GX7.3 microarray software.

### Artificial neural network (ANN) for diagnosis

The neural-network design consisted of a three-layer network: an input layer, with 13 units containing the information on the diagnostic criteria; a hidden layer, with 7 units; and an output layer for the detection of PD based on stages. A NeuNet Pro software (CorMac Technologies Inc., Canada), back prop algorithm were used for the prediction of disease stages. Back prop algorithm of NeuNet Pro software accepts the clinical data as numerical inputs. The training variables include age, gender, amplitude (si) of 10 NMR peaks (1.32 ppm, 1.46 ppm, 2.55 ppm, 2.60 ppm, 2.70 ppm, 3.10 ppm, 3.22 ppm, 3.54 ppm, 3.86 ppm, 4.02 ppm) representing ~292 metabolites and, together with information about disease status, normal (coded 0), stage1 (coded 1), stage2 (coded 2), stage3 (coded 3). The patterns of input facts associated with diagnoses were trained with randomly selected 45 individuals from the set 80 with known pathological status which includes 17 normal and 28 PD patients (9 samples of stage 1, 8 samples of stage 2 and 11 samples of stage 3). The training process continued until the differences between the network classifications and the clinical diagnoses became acceptable. Once the network was trained, the remaining 35 individuals were "tested," by means of the trained network. The neural network classifications were then compared with the known clinical diagnoses, to see whether the network was able to classify disease status reliability.

## Results

Investigation of plasma samples for the detection of metabolite variations were performed by comparing the normal with PD patients by proton NMR. The typical analysis of NMR spectra aimed for 22 metabolites hypothetically suggests a critical role in PD.

### Metabolite variations

**Figure 1:**
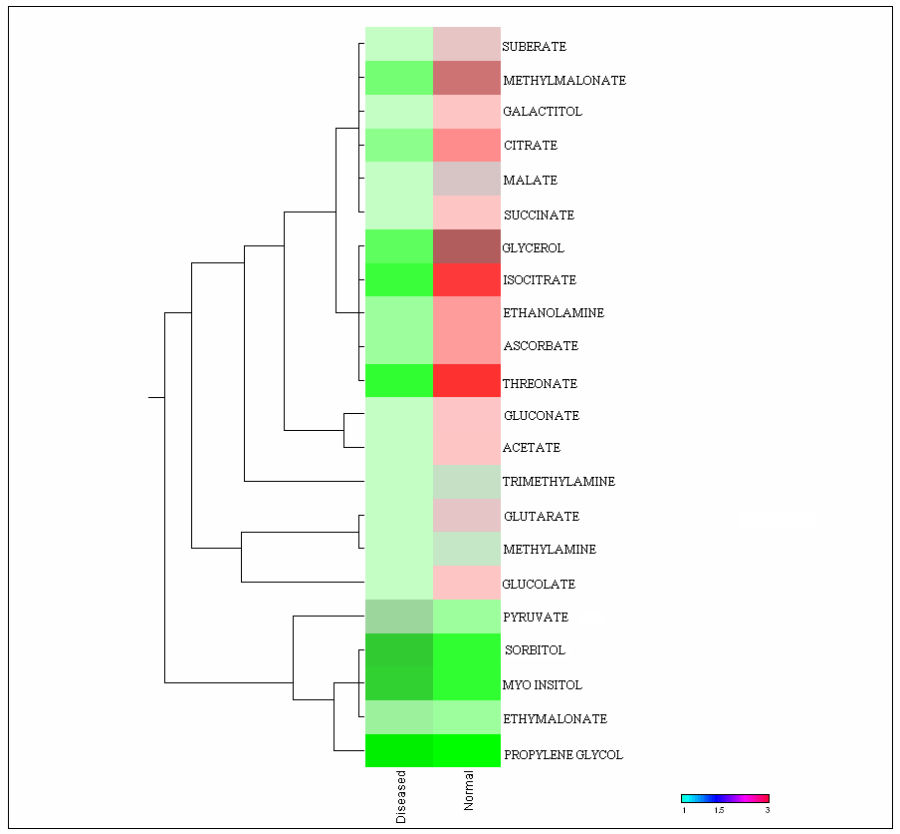
**Heat map differentiation of metabolite**. Average metabolite variability of blood plasma between PD patients (n = 43) and healthy controls (n = 37) are shown. Cluster analyses of the 22 differentially altered metabolites are selected based on significance *P *value (*P *< 0.05). The heat map depicts high (red) and low (green) relative levels of metabolite variation.

The mean concentrations of galactitol, glycerol, methylamine, trimethylamine, ethanolamine, suberate, glutarate, malate, methylmalonate, succinate, acetate, gluconate, threonate, glucolate, ascorbate, isocitrate, and citrate were significantly decreased, while the levels of ethymalonate, pyruvate, myoinositol, sorbitol and propylene glycol was elevated in the patient samples. The average difference in concentrations of metabolites between normal and patients was showed in heat map (Fig. 1).

### Statistical analysis

**Figure 2:**
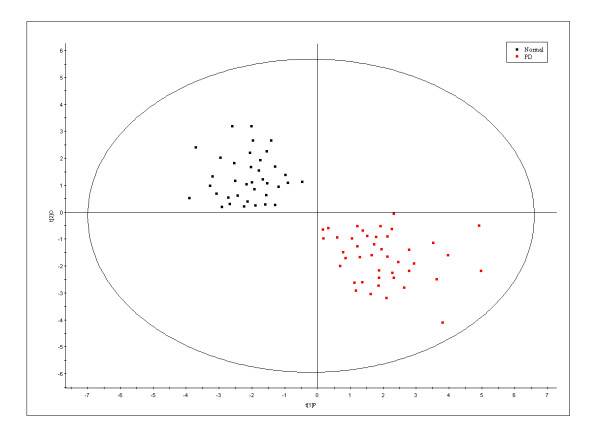
**Partial least square discriminant analysis**. PLS-DA scores plot showing a significant separation between control subjects (n = 37) and unmedicated PD patients (n = 43) using complete digital maps. The observations coded according to class membership: black square = controls; red square = PD patients.

**Figure 3:**
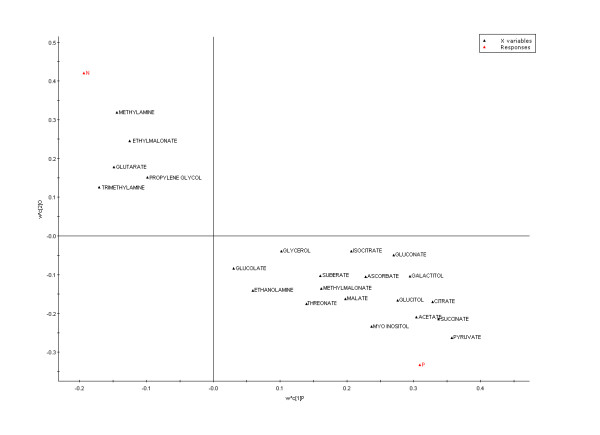
**Biomarker detection**. The loading coefficient map showing that myoinositol, glucitol, citrate, acetate, succinate and pyruvate were predominantly responsible for the classification of groups.

Analysis of variance (ANOVA) was performed to demonstrate a significant difference between PD patients and controls. The results showed the significance at p < 0.05 (Table 2). To explore the metabolite multidimensional data, unsupervised statistical methods were executed on samples. PLS-DA plots' scores based on ^1^H-NMR spectra of plasma samples showed a clear differentiation between healthy volunteers and drug-naive patients (Fig 2). The loading coefficient map indicates that myoinositol, glucitol, citrate, acetate, succinate and pyruvate were predominantly responsible for the separation between classes (Fig 3). Hence, the results from ^1^H-NMR spectroscopy showed significantly elevated concentrations of myoinositol, glucitol and pyruvate in plasma samples of drug-naive patients, confirming the likely importance of these molecules as biomarker.

**Table 2:** Statistical significance of metabolites

S. No	Metabolites	Significance (P < 0.05)
1	Glucitol	0.031

2	Galactitol	0.0420

3	Glycerol	0.0321

4	Methylamine	.0276

5	Trimethylamine	0.0318

6	Ethanolamine	0.0478

7	Suberate	0.0211

8	Glutarate	0.0210

9	Malate	0.133

10	Methylmalonate	0.0398

11	Succinate	0.0291

12	Acetate	0.0201

13	Gluconate	.0365

14	Threonate	0.0195

15	Glucolate	.0371

16	Ascorbate	0.0489

17	Isocitrate	0.0263

18	Pyruvate	0.0107

19	Citrate	0.0209

20	Ethylmalonate	0.0481

21	Myoinositol	0.0199

22	Propylene glycol	0.0429

Analysis of variance (ANOVA) of 22 metabolites showing the clear significant difference between PD patients and controls with p < 0.05

### Genetic aspects of pyruvate variation

**Figure 4:**
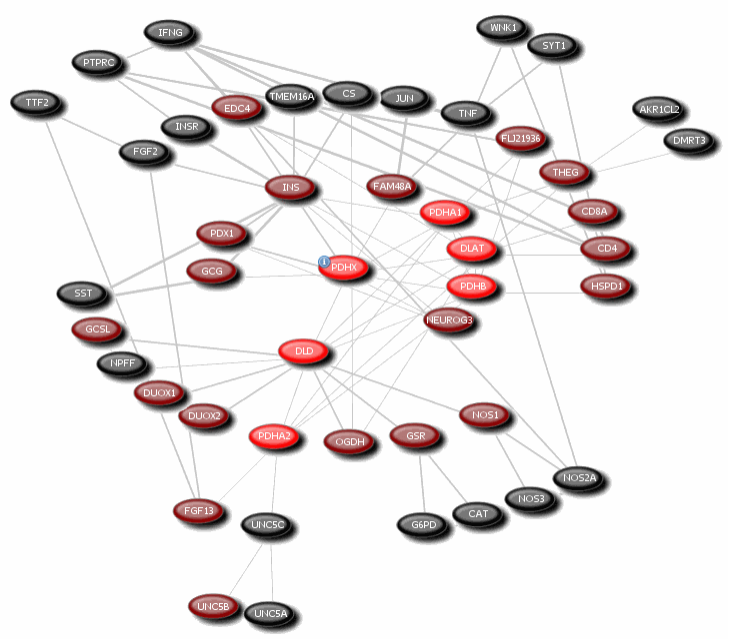
**Pyruvate dehydrogenase component interacting genes**. The systems biological approach showing the complex interaction of pyruvate dehydrogenase components (red) with 40 genes.

To determine the genetic basis of pyruvate variation, the systems biological approach was performed using PUBGENE index on *DLAT, DLD, PDHX, PDHB, PDHA1 *and *PDHA2 *genes. The result explores the interaction of 46 genes, such as *AKR1CL2, CAT, CD4, CD8A, CS, DMRT3, DUOX1, DUOX2, EDC4, FAM48A, FGF13, FGF2, FLJ21936, GCG, GCSL, G6PD, GSR, HSPD1, IFNG, INS, INSR, JUN, NEUROG3, NOS1, NOS2A, NOS3, NPFF, OGDH, PDX1, PTPRC, SST, SYT1, THEG, TMEM16A, TNF, TTF2, UNC5A, UNC5B, UNC5C *and *WNK1 *including pyruvate dehydrogenase components (Fig. 4). The biological significance of these genes in PD was further validated by secondary gene expression analysis. The expression analysis was executed only on 40 gene spots by excluding 6 genes such as *GCSL*, *FLJ21936, DMRT3, AKR1CL2, UNC5A *and *UNC5B*. The result shows the moderate down regulation of *CAT, PDHB, NPFF, TMEM16A, UNC5C, FGF13, JUN *and up regulation of *INSR, THEG, NOS1, FAM48A, PTPRC, OGDH, SYT1, FGF2 *and *SST *genes in cellular blood of PD. In addition, statistical analysis was carried out on 16 genes showed the significance of pyruvate dehydrogenase lipoamide beta (*PDHB*) and neuropeptide FF-amide peptide precursor (*NPFF*) genes with p < 0.05.

### Neural network classification

**Figure 5:**
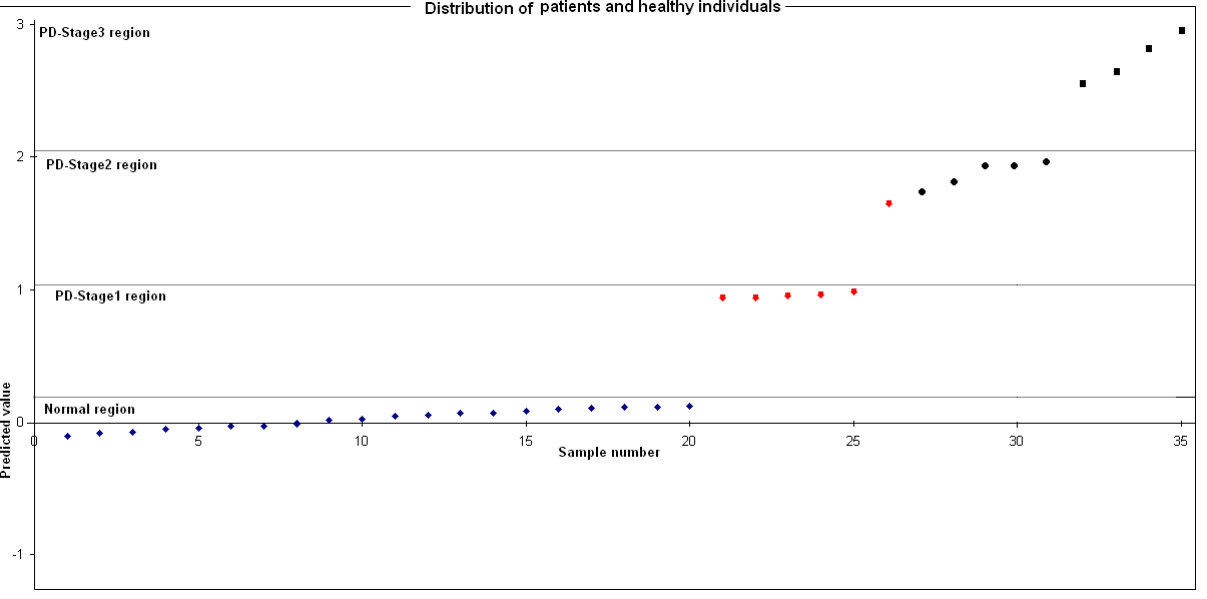
**Distribution of patients and healthy individuals**. Neural network classification of disease for 35 individuals (x-axis) with known clinical information. Values (y-axis) are predicted value over the trained network and are 0 to3; values ≥ 0.2 reflect a neural-network classification of "normal," and values ≤ 0.2 reflect a neural-network classification of "PD". Individuals denoted by a Violet rhombus are normal, red arrow are clinically stage 1 PD, blackened circle are stage 2, blackened square are stage 3 PD respectively.

The neural network training was performed as described above, on the basis of diagnostic criteria for the 45 individuals of known variable information. The network, tested with 35 individuals, the success rate in classification of the test set was 97.14% (34/35) accuracy, the sensitivity was 93.33% (14/15, one case of stage 1 was misdiagnosed as stage 2 PD), the specificity was 100% (20/20) (Fig. 5). Moreover, the values generated by the neural network were ranged from 0 to 3. On the basis of these values, individuals were assigned as affected or normal group. Individuals whose final predicted values were ≤ 0.2 were assigned to the normal group, and those whose output values ≥ 0.2 were assigned to the PD. For instance the predicted range from < 0.2 > 1.03 assigned as stage 1, ≤ 1.03 > 2.06 as stage 2 and the values ≤ 2.06 ≥ 3 as stage 3 (Table 3).

**Table 3:** Neural network prediction

Sample ID	ANN classification values	Neural network designation	Clinical designation
2	-0.085	Normal	Normal

3	-0.065	Normal	Normal

4	-0.045	Normal	Normal

6	-0.04	Normal	Normal

7	-0.02	Normal	Normal

8	-0.01	Normal	Normal

10	-0.01	Normal	Normal

11	0	Normal	Normal

12	0.02	Normal	Normal

13	0.03	Normal	Normal

14	0.052	Normal	Normal

15	0.059	Normal	Normal

20	0.073	Normal	Normal

21	0.078	Normal	Normal

22	0.092	Normal	Normal

29	0.105	Normal	Normal

31	0.112	Normal	Normal

32	0.118	Normal	Normal

34	0.122	Normal	Normal

35	0.1255	Normal	Normal

18	0.97	PD-Stage 1	PD-Stage 1

23	0.97	PD-Stage 1	PD-Stage 1

24	0.98	PD-Stage 1	PD-Stage 1

26	0.99	PD-Stage 1	PD-Stage 1

1	1.01	PD-Stage 1	PD-Stage 1

27	1.531	PD-Stage 2	PD-Stage 1*

5	1.62	PD-Stage 2	PD-Stage 2

17	1.73	PD-Stage 2	PD-Stage 2

25	1.97	PD-Stage 2	PD-Stage 2

28	1.97	PD-Stage 2	PD-Stage 2

30	1.99	PD-Stage 2	PD-Stage 2

9	2.46	PD-Stage 3	PD-Stage 3

16	2.56	PD-Stage 3	PD-Stage 3

19	2.82	PD-Stage 3	PD-Stage 3

33	2.96	PD-Stage 3	PD-Stage 3

*Sample ID: Misclassified stage 1 as stage 2 by ANN

Neural network classification values, neural network designation and disease designation, for 35 Individuals who have known clinical information. Individuals denoted by a (*) indicates the misclassification of PD stage1 as stage2 by ANN.

## Discussion

The present study is focused to identify the metabolic marker for the detection of PD from plasma and to validate the variations by spotting out through gene responsibility.

Several studies have been indicated the gene variation in PD [24-28] which may lead to metabolic abnormalities that are detectable in peripheral tissues. Few successful break through have shown metabolite variations in CSF and serum samples of PD. For instance, glutamate was found to be decreased in parkinsonian CSF compared to control subjects, a result reported in earliest analyses of parkinsonian CSF [29-31]. Subsequent study shows that the increased level of glycine, aspartate and glutamate in the plasma of parkinsonian patients [32]. Increase in CSF glycine also been observed in PD [33]. Previous study of PD patients showed reduction in concentrations of arginine and methionine in serum, while the level of valine was increased [34]. Additionally, a significant decrease in CSF isoleucine, alanine, lysine and moderate increase of a glutamine level was very well observed in PD [34]. Recent study shows the feasibility of potential diagnosis of PD from the detection of increased 8-OHdG in serum and urine of PD [35]. Furthermore, several reports indicate that there is a reduction of complex 1 activity in the electron transport chain of PD [36-41]. From the detailed knowledge of these studies, 22 metabolites, which play a role in mitochondrial function and other related pathway, have been targeted.

Analysis of the ^1^H NMR spectra of plasma samples showed differential distribution of these metabolites in drug-naive patients compared to the healthy volunteers. The targeted metabolite profiling of 22 compounds in blood plasma was characteristically altered in patients with PD, and most of these metabolites have decreased in concentration. The results showed that, this approach has great hope in diagnosis of PD. Moreover, this study was made with unmedicated PD patients to controls, to avoid the confounding effects of any medications. The key metabolites, such as myoinositol, sorbitol, citrate, acetate, succinate and pyruvate are significant in contribution for the separation between metabolite profiles of unmedicated PD patients and controls.

Plasma myoinositol level was significantly increased in the drug-naive patients. Elevation of myoinositol concentrations implies the decrease in activity of sciatic motor-nerve conduction velocity determined in animal models [42,43]. Elevated plasma myoinositol has not previously been reported for PD. However, studies show the abnormal myoinositol levels in the brain of neurologically diseased patients and other disorders [44-47]. Raised myoinositol levels in the basal ganglia were recently observed in PD patients under exercise condition detected using magnetic resonance spectroscopy [48]. Additionally, plasma sorbitol level was significantly increased in drug-naive patients. Sorbitol has been linked to drug treatment in PD [49], yet our observation of an elevation of plasma sorbitol concentrations in drug-naive patients may be due to impairment in oxidative stress [50,51]. The elevated levels of sorbitol have been reported in the CSF of mood disorder patients, which relates to oxidative stress [52]. Surprisingly, elevation in the plasma sorbitol level has not been reported in PD to our knowledge. Together with the significant findings of myoinositol and sorbitol in plasma imply the dysfunction of polyols metabolic pathway. Polyols pathway is a minor metabolic pathway of glucose running parallel to glycolysis, whose activity is altered in mitochondrial dysfunction [53]. However, malfunctioning of mitochondria is previously reported in PD [36-41], and it is the pedestal of this study. Detection of glucose concentration were not been carried out but abnormality of glucose metabolism was previously reported in PD patients [54].

More interestingly citrate, malate, acetate, succinate and pyruvate are significantly varied in PD plasma samples, contributes to the major distinction of PD from normal samples in PLS-DA analysis. These metabolites, such as citrate, acetate, succinate and malate were decreased, while increase in pyruvate concentration was noticed. Pyruvate is the end metabolite of glycolysis. It enters Kreb's cycle as acetyl-coA by the catalysis of enzyme pyruvate dehydrogenase in the presence of the coenzyme NAD+. The accumulation of pyruvate or its increased concentration in plasma may be due to abnormal activity of pyruvate dehydrogenase complex and its interacting genes in patients. Increased pyruvate CSF has already been reported in Alzheimer's patients [55,56]. The other intermediates of Kreb's cycle such as citrate, malate and succinate were considerably decreased in this study, which may correlate to alteration of pyruvate dehydrogenase activity. Moreover, the detection of other metabolites of Kreb's cycle was not executed in our analysis. Systems biological approach was carried out on pyruvate dehydrogenase components to identify its interacting genes which are hypothesized as the cause for the increased plasma pyruvate concentration. The analysis reveals 46 interacting genes together with pyruvate dehydrogenase components.

The significant variation in plasma pyruvate was validated by gene expression analysis of 46 genes derived from systems biological approach. The expression analysis was executed only on 40 genes and the other 6 genes in which the following FLJ21936, DMRT3, AKR1CL2, UNC5A and UNC5B were excluded from this study, because these genes are not expressed in blood cells and GCSL gene was also eliminated since it is considered as an alias of DLD gene. The analysis of 40 genes shows differential regulation of 16 genes in comparison to control. Interestingly, out of 16 genes, 9 genes have been previously reported in PD, such as CAT, FGF13, JUN, INSR, NOS1, OGDH, SYT1, FGF2 and SST [57-65]. Furthermore, statistical analysis of these genes show the significance of NPFF and PDHB with p < 0.05. NPFF gene plays a major role in inflammation modulation, neuroendocrine function and cardiovascular regulations [66], where as, PDHB gene is the beta subunit of pyruvate dehydrogenase, directly associated with pyruvate dehydrogenase activity and mitochondrial dysfunction. Abnormal activity of pyruvate dehydrogenase is the basis of our study in representing the variation in pyruvate concentration. The impairment of cardiovascular regulation [67], inflammation [68] and neuroendocrine function [69] were reported in PD and this may suggest the influence of differential regulation of NPFF gene. The biological significance of these genes related to PD has not been reported previously. Hence, the variation in pyruvate concentration can be considered as a marker for detection of PD and future studies need to be carried out on the factors and the hidden mechanism involved in variations of NPFF and PDHB in PD.

Prior study indicates no correlation between metabolite variation with severity and duration in PD [24]. To bring out the correlation, NMR peak amplitude representing the ~290 metabolite was included as one of the variables in the prediction of disease stages by neural network. The ANN was trained with variable parameters as described above. More fascinating results emerged from neural network and it is capable to predict early stages of PD, with a good accuracy using these variables. The optimized network yield has an accuracy of 97.14% in detecting PD patients. Based on the present study it has been confirmed that the association between disease progression and metabolite variation is strong and it is more accurate than the current diagnosis [23] of PD.

## Conclusion

The present study concludes that the application of NMR metabolite profiling of plasma fluid can provide an efficient means for detection of Parkinson's disease. We identified abnormalities in 22 circulating metabolites, which bring out the scope for the diagnosis of PD. Moreover, the result obtained from neural network approach is more feasible for the stage wise detection. However, the accuracy of neural network is questionable unless the study focused with the larger samples.

## Competing interests

The authors declare that they have no competing interests.

## Authors' contributions

SSSJA designed the study and analyzed the data under the guidance of HTTC and WS. The research participants were selected by SK, examined them clinically and neurologically for metabolic investigations.
